# Nutrients Differentially Regulate Nucleobindin-2/Nesfatin-1 *In Vitro* in Cultured Stomach Ghrelinoma (MGN3-1) Cells and *In Vivo* in Male Mice

**DOI:** 10.1371/journal.pone.0115102

**Published:** 2014-12-15

**Authors:** Haneesha Mohan, Naresh Ramesh, Sima Mortazavi, Anthony Le, Hiroshi Iwakura, Suraj Unniappan

**Affiliations:** 1 Laboratory of Integrative Neuroendocrinology, Department of Veterinary Biomedical Sciences, Western College of Veterinary Medicine, University of Saskatchewan, Saskatoon, Saskatchewan, Canada; 2 Department of Biology, York University, Toronto, Ontario, Canada; 3 Medical Innovation Center, Kyoto University Graduate School of Medicine, Sakyo-ku, Kyoto, Japan; University of California, Los Angeles, United States of America

## Abstract

Nesfatin-1 is secreted, meal-responsive anorexigenic peptide encoded in the precursor nucleobindin-2 [NUCB2]. Circulating nesfatin-1 increases post-prandially, but the dietary components that modulate NUCB2/nesfatin-1 remain unknown. We hypothesized that carbohydrate, fat and protein differentially regulate tissue specific expression of nesfatin-1. NUCB2, prohormone convertases and nesfatin-1 were detected in mouse stomach ghrelinoma [MGN3-1] cells. NUCB2 mRNA and protein were also detected in mouse liver, and small and large intestines. MGN3-1 cells were treated with glucose, fatty acids or amino acids. Male C57BL/6 mice were chronically fed high fat, high carbohydrate and high protein diets for 17 weeks. Quantitative PCR and nesfatin-1 assays were used to determine nesfatin-1 at mRNA and protein levels. Glucose stimulated NUCB2 mRNA expression in MGN3-1 cells. L-Tryptophan also increased NUCB2 mRNA expression and ghrelin mRNA expression, and nesfatin-1 secretion. Oleic acid inhibited NUCB2 mRNA expression, while ghrelin mRNA expression and secretion was enhanced. NUCB2 mRNA expression was significantly lower in the liver of mice fed a high protein diet compared to mice fed other diets. Chronic intake of high fat diet caused a significant reduction in NUCB2 mRNA in the stomach, while high protein and high fat diet caused similar suppression of NUCB2 mRNA in the large intestine. No differences in serum nesfatin-1 levels were found in mice at 7 a.m, at the commencement of the light phase. High carbohydrate diet fed mice showed significantly elevated nesfatin-1 levels at 1 p.m. Serum nesfatin-1 was significantly lower in mice fed high fat, protein or carbohydrate compared to the controls at 7 p.m, just prior to the dark phase. Mice that received a bolus of high fat had significantly elevated nesfatin-1/NUCB2 at all time points tested post-gavage, compared to control mice and mice fed other diets. Our results for the first time indicate that nesfatin-1 is modulated by nutrients.

## Introduction

Nesfatin-1 [NEFA/NUCB2-encoded satiety and fat-influencing protein-1] is a potent anorexigenic peptide implicated in the regulation of energy balance and glucose homeostasis [1], [30]. It is an 82 amino acid peptide derived from the precursor protein, nucleobindin-2 (NUCB2) [1]. NUCB2 is composed of 396 amino acids, consisting of two EF hand motifs and a DNA binding domain [1], [3]. Post-translational processing by prohormone convertases (PC 1/3 and PC 2) causes NUCB2 to be cleaved into three peptides, nesfatin-1 (1–82 amino acids), nesfatin-2 (85–163 amino acids), and nesfatin-3 (166–396 amino acids). NUCB2/nesfatin-1 amino acid sequence is highly conserved across vertebrates [6], [7], [31]. NUCB2/nesfatin-1 is found in various hypothalamic nuclei that are involved in energy metabolism, such as the arcuate nucleus, paraventricular nucleus, supraoptic nucleus, lateral hypothalamic area and zona increta [8], [9]. Insulin producing beta cells co-express nesfatin-1 in the pancreatic islets of rats and mice [2], [4], [11], suggesting that nesfatin-1 could play an important role in insulin secretion and glucose homeostasis [4], [29]. Ghrelin and NUCB/nesfatin-1 are colocalized in the gastric oxyntic mucosal glands in rodents [13] and humans [16]. NUCB2 mRNA expression in purified gastric mucosal endocrine cells was found to be higher than in the brain of rats [13]. The full length NUCB2 protein was observed in the small and large intestines and liver of male rats, and ICR mice [5]. The wide distribution of NUCB2/nesfatin-1 in central and peripheral tissues points to a role for nesfatin-1 in regulating metabolism.

Daily administration of nesfatin-1 caused extended reduction in food intake and body weight [1]. Intracerebroventricular administration of NUCB2 suppresses food intake, body weight and subcutaneous, mesenteric and epididymal fat mass in adult rats in a dose dependent manner. In addition, NUCB2 knockdown in rats by infusing antisense morpholino oligonucleotide (as-MON) caused an increase in appetite and body weight [1]. Intra-paraventricular nucleus injection of nesfatin-1 reduces cumulative food intake at 1 and 3 hours [9]. Intraperitoneal injections of nesfatin-1 resulted in a reduction in food intake in leptin resistant *db/db* mice and high fat diet fed mice [8]. Nesfatin-1 is composed of 3 structural fragments and only the mid-fragment (residues 24–53; M30) of nesfatin-1 is involved in producing anorectic responses [15], [28]. Together, these results provide clear evidence that support satiety effects of nesfatin-1.

Nesfatin-1 is a meal responsive glucoregulatory hormone [12], [13], and pancreatic islets of rats release NUCB2 in response to glucose [14]. In human studies, glucose treated subjects had higher basal nesfatin-1 levels compared to control subjects [10]. In MIN6 cells, a 4-fold increase in nesfatin-1 levels was observed when the cells were incubated in high glucose (16.7 mM) compared to low glucose (2.0 mM) [4]. Nesfatin-1 enhanced glucose stimulated insulin secretion from cultured MIN6 cells that were incubated in high glucose than in low glucose in a dose dependent manner [4]. In the pancreas of streptozotocin (STZ)-injected mice with Type 1 Diabetes, it was found that both NUCB2 and preproinsulin mRNA expression were significantly lower [4]. In contrast, enhanced nesfatin-1 co-localization with insulin was found in the islet beta cells of high-fat diet-induced obese mice with Type 2 Diabetes. Nesfatin-1 has tissue specific effects on glucose uptake in rat adipocytes and muscle [30]. Overall, nesfatin-1 exerts important roles in regulating whole body glucose and energy homeostasis.

While nesfatin-1 is emerging as an important meal responsive peptide [1], [12], [13], [30], what triggers its secretion remains unclear. What diet components trigger the post-meal secretion of nesfatin-1? This question remains unaddressed. The main focus of this study is to determine how different nutrients can modulate NUCB2/nesfatin-1 *in vitro* in cultured stomach ghrelinoma (MGN3-1) cells from mice and *in vivo* in male mice. Our results from our *in vitro* studies indicate that MGN3-1 cells respond differently to nutrients in secreting NUCB2/nesfatin-1 and ghrelin. Similarly, acute or chronic intake of nutrients does influence NUCB2 mRNA expression and NUCB2/nesfatin-1 release in a diet specific manner.

## Materials and Methods

### Ethics Statement

All studies using animals complied with the Canadian Council of Animal Care guidelines, and were approved by the Animal Research Ethics Board of the University of Saskatchewan (Protocol Number 2012-0033).

### *In Vitro* Studies

Mouse stomach ghrelinoma (MGN3-1) cells [17] were cultured in DMEM (Invitrogen, Ontario, Canada; Catalogue #11995-040) that was supplemented with 10% fetal bovine serum (Invitrogen; Catalogue #12484) and 1% penicillin (100 U/mL) and streptomycin (100 µg/mL) (Invitrogen; Catalogue #15140-122) at 37°C in 10% CO_2_. At 80% confluency, MGN3-1 cells were seeded at 6×10^6^ cells/well in a 12-well plate and the studies were performed when cells were 80–90% confluent. Each study was repeated thrice and the data from three studies were pooled to obtain an n = 9–12 wells/treatment. To determine whether glucose had an effect in a dose and time dependent manner, cells were incubated for 1 hour and 2 hours with 5.6, 25, 50, and 100 mM glucose DMEM media. The complete growth medium of MGN3-1 cells requires them to be growing at a high glucose level, which is 25 mM. In relation to the studies conducted with fatty acids and amino acids, we performed these studies using DMEM at low glucose levels (5.6 mM), since using a high glucose medium (25 mM) could mask the effect of the respective nutrients on NUCB2/nesfatin-1 secretion and synthesis. With respect to long chain fatty acids, we tested the effect of three different fatty acids using linolenic acid (Sigma-Aldrich, Ontario, Canada; Product #L2376), octanoic acid (Sigma-Aldrich; Product #C2875) and oleic acid (Sigma-Aldrich; Product #O1383). The cells were incubated for 4 hours with each fatty acids at 0, 1, 10, 100 µM. We used L-Tryptophan (Sigma-Aldrich; Product #T8941) to test the effect of an amino acid on NUCB2 secretion and synthesis. The cells were incubated for 4 hours with L-tryptophan at 0.7, 1, 10 mM. L-Tryptophan is present in the control medium (5.6 mM glucose DMEM) at a minimum dose of 0.7 mM, which is essential for their growth condition.

### *In Vivo* Studies

For the chronic feeding of diets containing varying amounts of specific nutrients, age and weight-matched (5 weeks old, average body weight: 20 grams) male C57BL/6 mice (Charles River Laboratories, Quebec, Canada) were housed individually for 17 weeks in a 12 hours light: 12 hours dark cycle (lights off at 7 PM and on at 7 AM), temperature and humidity controlled vivarium. Mice were divided into four groups fed on a control (n = 6), high carbohydrate (n = 7), high protein (n = 7), and high fat (n = 7) diet with *ad libitum* access to water and their specific diet. All diets were purchased from Research Diets (New Brunswick, NJ). The calorie content of diets were: control (Product #D12451): 4.73 kcal/gm with 20% energy derived from protein, 35% energy derived from carbohydrate and 45% energy derived from fat; high carbohydrate (Product #D12450J) had 3.8 kcal/gm with 20% energy derived from protein, 70% energy derived from carbohydrate and 10% energy derived from fat; high protein (Product #D08091802) had 3.8 kcal/gm with 60% energy derived from protein, 30% energy derived from carbohydrate and 10% energy derived from fat, and high fat (Product #D12492) had 5.2 kcal/gm with 20% energy derived from protein, 20% energy derived from carbohydrate and 60% energy derived from fat. All mice were fed with the control diet for one week prior to starting their specific diets. Food intake, body weight, and blood glucose readings post 4 hours fast were measured once a week for 17 weeks.

For the acute administration of nutrients, age and weight-matched (5 weeks old, average body weight: 20 grams) male C57BL/6 Mice (Charles River Laboratories, St Constant, QU, Canada) were housed individually for 1 week and 2 days in a 12 h light:12 h dark cycle (lights off at 7 PM and on at 7 AM), temperature and humidity controlled vivarium. Mice were acclimatized for 1 week upon arrival and had ad libitum access to water and regular mouse chow for 11 days. Since we are performing an acute diet study, we needed the animals to acclimatize to the oral gavage procedure. We acclimatized the mice to this procedure by gavaging them with tap water for 2 days prior to the experimental day. On the 12^th^ day, the mice were fasted for 4 hours and were gavaged with a specific liquid diet. The mice were divided into 4 groups: High protein (Isopure Protein Drink, Zero Carb - Mango peach flavor; Nature’s Best, Clifton Park, New York; n = 7), High fat (Splendido; Cold Pressed Extra Virgin Olive oil; President’s Choice, Canada; n = 7), High carbohydrate (D-Glucose; BioShop; Catlogue #GLU501.500; n = 7), and Water (tap water; n = 7). On the day of the study, 200 microliters of the above nutrients/water was administered to the mice by oral gavage. Blood glucose readings was taken at 0, 5, 10, 15, 20, 30, 60, 90 and 120 minutes, and blood was collected at 15, 30, 60, and 120 minutes for ELISA analysis to determine circulating levels of NUCB2/nesfatin-1. Tissues (stomach, small intestine [duodenum], large intestine and liver) were collected from each mouse upon termination of the study (deep isoflurane euthanasia followed by cervical dislocation). To maintain consistency, the timing and the duration of each experiment, surgeries and sample collection were kept constant for all studies.

### Total RNA Extraction and cDNA Synthesis

Cells or tissues were collected from each study to compare NUCB2 mRNA expression. From the mice that underwent the chronic diet study, tissues (stomach, small intestine, large intestine and liver) were harvested immediately after euthanasia. Total RNA was extracted from the MGN3-1 cells and tissues, using the TRIzol RNA isolation reagent (Invitrogen). RNA purity was validated by optical density (OD) absorption ratio (OD 260 nm/OD 280 nm) using a NanoDrop 2000c (Thermo, Vantaa, Finland). Only samples with an absorption ratio greater than 1.8 were used for cDNA synthesis. Synthesis of cDNAs was conducted using iScript cDNA synthesis kit as directed by the manufacturer (BioRad, Canada).

### RT-PCR and Quantitative Real Time-PCR

RT-PCR and qRT-PCR for NUCB2, ghrelin, and RT-PCR for PC 1/3 and PC 2 were conducted as per conditions outlined in **Table 1**, using the CFX Connect Real-Time PCR Detection System (Bio-Rad). For the qRT-PCR analysis, mRNA expression of NUCB2 was normalized using beta-actin as a housekeeping gene. PCR products for NUCB2 in the stomach, liver and large intestine and these genes (NUCB2, ghrelin, PC 1/3 and PC 2) in the MGN3-1 cells were electrophoresed in 1% agarose gel to verify transcripts amplified. Based on previous studies [4], we used beta-actin as an internal control to normalize the signal of NUCB2 mRNA. When using total RNA where mRNA quantification was very precise, the critical threshold values for beta-actin showed no variability. Relative NUCB2 mRNA expression was normalized with beta-actin from the same sample according to the Livak method [31].

**Table 1:** Sequences of forward and reverse primers, and the conditions employed in PCR and qRT-PCR analyses of the expression of mRNAs of interest.

Gene	Primers - Sequence (5′ to 3′)		PCR conditions [temperature (°C)/time (s)]
	Forward	Reverse	
**NUCB2**	CCAGTGGAAAATGCAAGGAT	GCTCATCCAGTCTCGTCCTC	35 cycles of 95°/10; 60°/30; 73°/30
**Ghrelin**	GCATGCTCTGGATGGACATG	CCTGATCTCCAGCTCCTC	35 cycles of 95°/10; 50.5°/30; 73°/30
**PC1/3**	AGTGGAAAAGATGGTGAATG	CTCCTCATTTAGGATGTCCA	35 cycles of 95°/10; 48.1°/30; 73°/30
**PC2**	AATGGGAGGAAGAGGAATC	TTGTTTTGAGGGTCAGTACC	35 cycles of 95°/10; 50.5°/30; 73°/30

### Immunocytochemistry and Microscopy

MGN3-1 cells were cultured in a Labtek Chamber Slide System (Nalge Nunc International, Rochester, NY) and were allowed to grow to near confluency. Cells were washed with 1X phosphate buffer solution (PBS; 2×5 minutes, 25°C) and fixed in a 4% paraformaldehyde (PFA) solution in 1X PBS for 10 minutes at 25°C, followed by another wash with 1X PBS (3×5 minutes, 25°C). The fixed cells were permeabilized in a solution of 0.3% Triton-X (Bioshop, Burlington, Ontario, Canada) in 1X PBS for 5 minutes at room temperature. Slides were incubated in blocking buffer containing 10% goat serum in 1X PBS for 1 hour at room temperature. Cells were then incubated in primary antibody **(** **Table 2** **)** at 4°C overnight. Slides were washed with PBS (3×5 minutes, 25°C) and incubated with secondary antibody (**Table 2** **;** the PC1/3 antibody was a generous gift from Dr. Iris Lindberg, University of Maryland School of Medicine) for 4 hours at room temperature. Finally, slides were washed with PBS (3×5 minutes, 25°C) and mounted with Vectashield mounting medium containing 4′, 6-diamidino-2-phenylindole (DAPI) (Vector Laboratories, Burlington, Ontario, Canada).

**Table 2:** Antibodies used for immunofluorescence microscopy.

Antibody	Dilution*	Raised In	Manufacturer/Source
**Ghrelin**	1∶100	Mouse	Abcam, Cambridge, MA, USA
**Nesfatin-1**	1∶100	Rabbit	Phoenix Pharmaceuticals, Burlingame, CA, USA
**PC1/3**	1∶100	Rabbit	Dr. Iris Lindberg, University of Maryland, USA
**PC2**	1∶100	Rabbit	Abcam, Cambridge, MA, USA
**Anti-Rabbit (Texas Red)**	1∶100	Goat	Vector Laboratories, Burlington, ON, Canada
**Anti-Mouse (FITC)**	1∶100	Goat	Abcam, Cambridge, MA, USA

*Antibodies were diluted in 1% bovine serum albumin.

Cells were viewed using a Nikon Eclipse-Ti inverted fluorescence microscope (Nikon, Mississauga, Ontario, Canada) and images were captured using a Nikon DS-Qi1 MC camera (Nikon). Images were analyzed using NIS-Elements basic research software (Nikon) on a Dell HP Workstation. Images shown are representative cells stained for ghrelin, NUCB2, PC 1/3 and PC2. For high resolution imaging, the cells were viewed, analysed and images captured using a Leica TCS SP5 confocal microscope.

### Western Blot Analysis, Immunohistochemistry and Fluorescence Microscopy

For confirming the presence of nucleobindin-2 (NUCB2) in the intestine and liver, three, 3 months old C57BL/6 male mice were used. Briefly, liver, and small and large intestines were collected, and separated for Western analysis or immunohistochemistry. Tissues for Western blot homogenized in T-PER tissue protein extraction reagent (Thermo Scientific, #78510) followed protein concentration determination by Bradford assay. The samples were prepared in 1X Laemmli buffer containing 0.2% 2-mercaptoethanol (Bio-Rad, #161-0737 and -0710) and subsequently were boiled at 95°C for 5 min followed by vortexing. The whole sample volume (20 µL) each containing 50 µg protein or synthetic rat nesfatin-1 (ABGENT, 1µg/µL; previously used in 4, 29) was loaded on a gel, and run in a Mini-PROTEAN TGX 8–16% gradient gel (Bio-Rad, #456-1104). After separation, the proteins were transferred to a 0.2 µm BioTrace nitrocellulose membrane (PALL Life Sciences, #27377-000) and then membrane was blocked in 1X RapidBlock solution (AMRESCO, #M325). NUCB2 protein detection was performed using rabbit anti-nesfatin-1 (Catalogue number H-003-22; 1∶500 dilution; Phoenix Pharmaceuticals, California) and GAPDH protein was detected by use of rabbit antiserum directed against mouse GAPDH (AbDSerotec, #AHP1628) diluted 1∶1000. As secondary antibody, goat anti-rabbit IgG (H+L) HRP conjugate (Bio-Rad, #170-6515) diluted 1∶3000 was used. For protein visualization the membrane was incubated for 5 min in Clarity Western ECL substrate (Bio-Rad, #170-5061) and imaged using ChemiDoc MP imaging system (Bio-Rad, #170-8280) with chemiluminescence detection. Membrane stripping in between protein detection was conducted using Restore PLUS western blot stripping buffer (Thermo Scientific, #46430). Precision plus protein dual xtra standards (Bio-Rad, #161-0377) were used as molecular weight markers.

For immunohistochemical studies, the tissues collected were fixed in 4% formaldehyde for 24 hours at 4°C. Fixative was replaced with ethanol (three 70% ethanol), each followed by a 10 minute incubation at 4°C. Tissues were then stored in 70% ethanol at 4°C and were processed and sectioned at the Prairie Diagnostic Services Inc. (PDS Inc., Western College of Veterinary Medicine, University of Saskatchewan). Paraffin sections of 4 µm thickness were prepared for immunostaining. These sections were deparaffinized with xylene (incubated twice in 100% xylene; 5 minutes, 25°C) and rehydrated in a graded ethanol series (incubated twice in 100% ethanol, and once in each 95% ethanol, 70% ethanol, 50% ethanol; 2 minutes each, 25°C). The sections were then incubated with 3% hydrogen peroxide in distilled water to block endogenous peroxidase activity (30 minutes at room temperature). The sections were then blocked with serum-free protein block reagent (DAKO Corporation, California) for 10 minutes before being incubated with primary antibodies. These sections were then incubated with rabbit anti-nesfatin-1 (Catalogue number H-003-22; 1∶500 dilution; Phoenix Pharmaceuticals, California) for 24 hours at room temperature. All slides were subsequently washed three times with 1x PBS and incubated with goat anti-rabbit Texas Red IgG (Red-Nesfatin-1; Catalogue number TI-1000; 1∶100 dilution; Vector Laboratories, California) secondary antibody for 1 hour at room temperature. All primary and secondary antibodies were diluted in antibody diluent reagent (DakoCytomation, Mississauga, Ontario). The slides were washed three times with 1x PBS and seven times with distilled water. Finally, the slides were mounted with Vectashield medium that contain nuclear dye DAPI (Blue; Vector Laboratories, Burlingame, California). Sections were viewed under a Nikon Eclipse Ti-E inverted fluorescence microscope (Nikon Canada, Mississauga, Canada). Images were captured using a Nikon DS-QI1 MC cooled monochrome camera connected to a Dell HP Workstation computer and NIS elements basic research imaging software (Nikon Canada, Mississauga, Canada). Only representative images of small and large intestine staining for NUCB2/nesfatin-1 with DAPI are shown in the results section.

### Nesfatin-1/NUCB2 Levels in Serum and Media

To investigate nutrient dependent changes in NUCB2/nesfatin-1 secretion from MGN3-1 cells, media was collected after specific incubation periods. In order to prevent cell debris, samples were centrifuged (13000 rpm for 10 minutes at 4°C) and the top 700 µL was stored at −20°C until nesfatin-1 measurement. For measuring circulating NUCB2/nesfatin-1, blood was collected at 7 a.m (soon after the light phase begins), 1 p.m. (middle of the light phase) and at 7 p.m (prior to the commencement of the dark phase). Blood samples were allowed to clot on ice, and serum was separated by centrifugation (7000 rpm for 9 minutes at 4°C) and stored at −20°C, until assays were conducted. NUCB2/Nesfatin-1 secretion levels in the media were measured using the Nesfatin-1 (1–82) (Rat) ELISA kit (Catalogue number EK-003-22, Phoenix Pharmaceuticals Inc., California). The limit of assay sensitivity was 1.2 ng/mL for nesfatin-1, with detectable range from 0.1–1000 ng/mL. The amount of immunoreactive material was determined using a non-linear regression curve-fit, which was used to quantify and compare the concentration of NUCB2/nesfatin-1 secretion in the serum and media samples.

### NUCB2/Nesfatin-1 Levels in Serum and Media and Total Ghrelin levels in media

To investigate nutrient dependent changes in NUCB2/nesfatin-1 and total ghrelin secretion from MGN3-1 cells, media was collected after specific incubation periods. In order to prevent cell debris, samples were centrifuged (13000 rpm for 10 minutes at 4°C) and the top 700 µL was stored at −20°C until NUCB2/Nesfatin-1 and total ghrelin measurement. Blood samples were allowed to clot on ice, and serum was separated by centrifugation (7000 rpm for 9 minutes at 4°C) and stored at −20°C, until assays were conducted. NUCB2/Nesfatin-1 secretion levels in the serum and media were measured using the Nesfatin-1 (1–82) (Rat) ELISA kit (Catalogue number EK-003-22, Phoenix Pharmaceuticals Inc., California). The limit of assay sensitivity was 1.2 ng/mL for nesfatin-1, with detectable range from 0.1–1000 ng/mL. Similarly, the total ghrelin secretion levels in the media was measured using the Ghrelin (Rat, Mouse) EIA kit (Catalogue number EK-031-31, Phoenix Pharmaceuticals Inc, California). The limit of assay sensitivity was 1.16 ng/mL for total ghrelin, with detectable range from 0–100 ng/mL. The amount of immunoreactive material was determined using a non-linear regression curve-fit, which was used to quantify and compare the concentration of NUCB2/nesfatin-1 secretion in the serum and media samples.

### Statistical Analysis

Analyses of the quantified qRT-PCR and ELISA data were conducted using One-Way ANOVA followed by Tukey’s multiple comparison test. GraphPad Prism version 5 (GraphPad Software Incorporated, San Diego, CA, USA) was used for statistical analyses and graphs. Significance was assigned when p<0.05. Data are expressed as mean ± SEM.

## Results

### NUCB2, PC 1/3 and PC 2 mRNAs are expressed in MGN3-1 cells and NUCB2 mRNA is expressed in the stomach, liver, small intestine and large intestine of male mice

**Figure 1:**
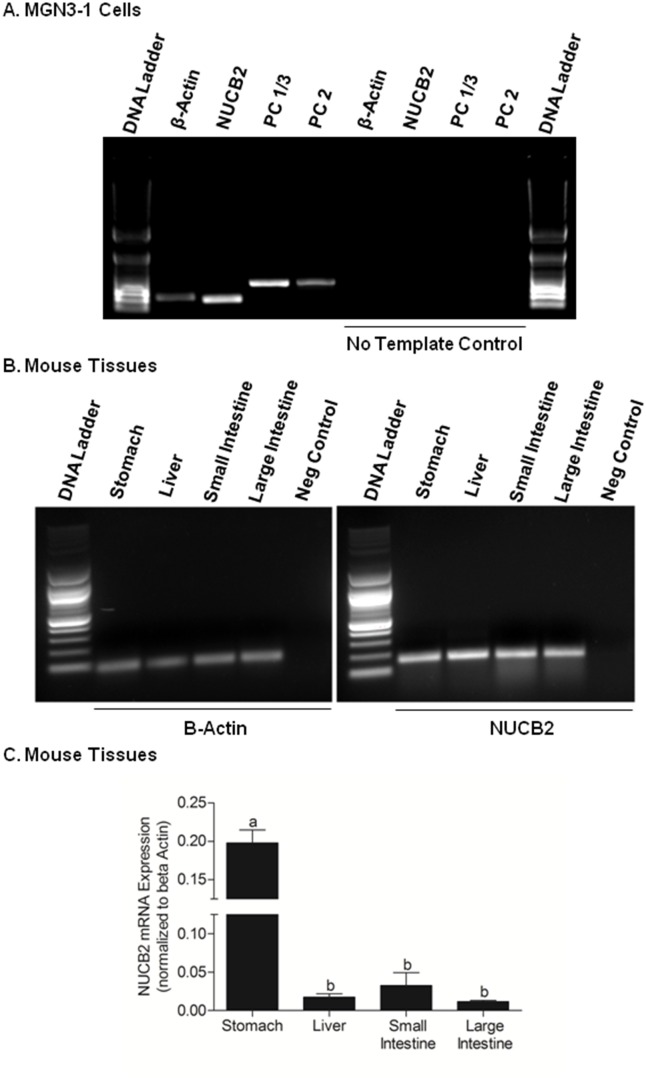
NUCB2, PC1/3 and PC2 mRNA Expression in MGN3-1 cells (A) and NUCB2 mRNA Expression in the Stomach, Liver, Small and Large Intestine from Male C57BL/6 Mice Tissues (B and C). Beta-Actin (β-Actin), nucleobindin-2 (NUCB2; 202 bp), prohormone convertase 1/3 (PC 1/3; 400 bp), and prohormone convertase 2 (PC 2; 406 bp) mRNAs were identified in MGN3-1 cells. No expression of these mRNAs was found in the RT-PCR reaction devoid of the cDNA template (A). Beta-Actin (β-Actin) and nucleobindin-2 (NUCB2; 202 bp) mRNAs expression were identified in the stomach, liver, small and large intestine from mice. No NUCB2 mRNA expression was found in PCR reactions devoid of the cDNA template (B). Relative abundance of NUCB2 mRNA expression normalized to β-Actin in the stomach, liver, small and large intestine is shown in (C). Letter “b” denotes significant difference from “a”, p<0.05, One-Way ANOVA followed by Tukey’s multiple comparison test.

We identified expression of NUCB2 (202 bp), prohormone convertase 1/3 (400 bp), and prohormone convertase 2 (406 bp) mRNAs in MGN3-1 cells **(** **Fig. 1A** **)**. NUCB2 (202 bp) mRNA expression was also detected in the stomach, liver, small intestine and large intestine of male C57/BL6 mice **(** **Fig. 1B** **)**. Absolute levels of NUCB2 mRNA expression in the stomach were higher than NUCB2 mRNA expression in the liver, small intestine and large intestine **(** **Fig. 1C** **)**.

### MGN3-1 cells are immunopositive for ghrelin, NUCB2/Nesfatin-1, PC 1/3 and PC 2

**Figure 2:**
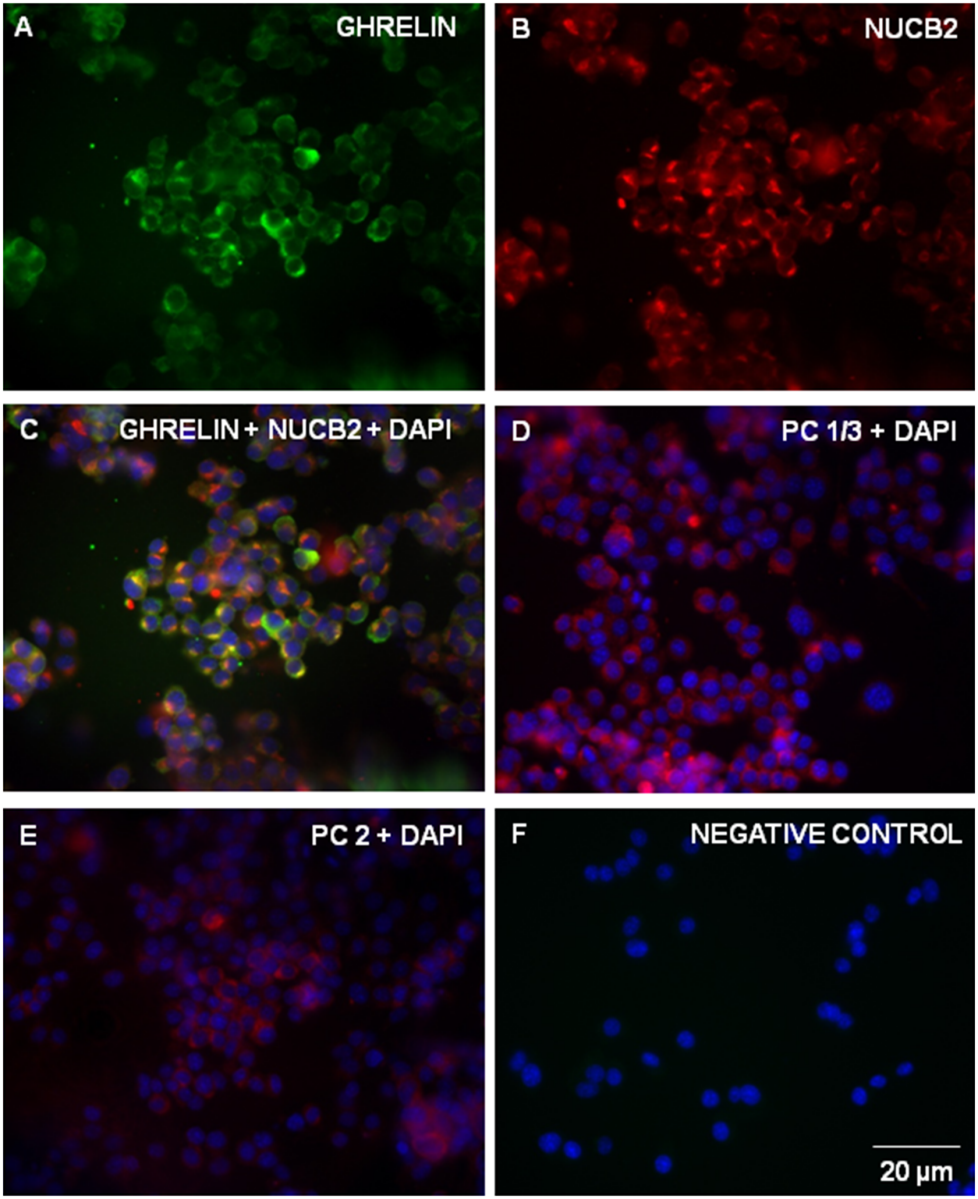
MGN3-1 Cells are Immunopositive for NUCB2/Nesfatin-1, Ghrelin, PC 1/3 and PC 2. Immunocytochemical staining of MGN3-1 cells for ghrelin immunoreactivity (A; FITC-Green), nesfatin-1 immunoreactivity (B; Texas-Red) and the nuclear stain DAPI. A merged image showing co-localization of nesfatin-1 and ghrelin immunoreactivity is shown in (C; Yellow). Immunocytochemical staining of MGN3-1 cells for PC 1/3 immunoreactivity (D; Texas-red), PC 2 immunoreactivity (E; Texas-red) and the nuclear stain DAPI. No primary antibody controls are shown in (F) for nesfatin-1 and ghrelin, respectively. Images taken at 40X magnification. Scale bar = 20 µm.

**Figure 3:**
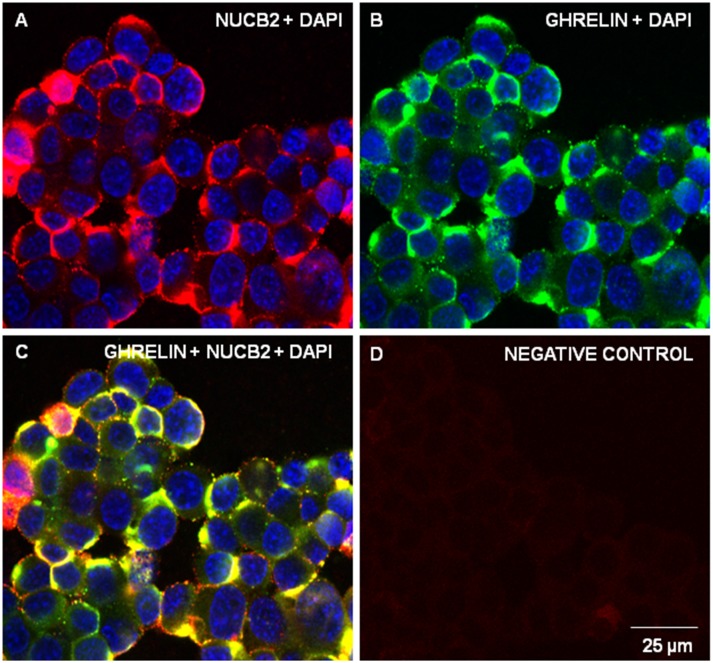
NUCB2/Nesfatin-1 co-localizes with ghrelin in MGN-3 cells. Confocal micrographs of MGN-3 cells stained for NUCB2/nesfatin-1 (A; Texas-Red) and ghrelin (B; FITC-Green). Merged image of A and B showing co-localization of NUCB2/nesfatin-1 and ghrelin immunoreactive cells (C; Yellow). No primary antibody negative control labeled only with secondary antibodies (D). Nuclei are stained with DAPI (Blue; A, B and C). Images were merged using Image J PC-based software. Scale bar = 25 µm.

Fluorescence microscopy displayed MGN3-1 cells stained with anti-nesfatin-1 antibody (Texas-Red; **Fig. 2B**) and anti-ghrelin antibody (FITC-Green; **Fig. 2A**) showed clear co-localization (Yellow; **Fig. 2C**) of nesfatin-1 and ghrelin immunoreactivity. However, some ghrelin positive cells were not immunoreactive for nesfatin-1 (**Fig. 2C**). MGN3-1 cells showed PC 1/3 immunoreactivity (Texas Red; **Fig. 2D**) and PC 2 immunoreactivity (Texas Red, **Fig. 2E**). DAPI (Blue) stained the nucleus of all cells including those cells not positive for the proteins studied. Control slides stained with secondary antibody alone (**Fig. 2F**) had no immunoreactivity. Confocal imaging showed MGN3-1 cells stained with anti-nesfatin-1 antibody (Texas-Red; **Fig. 3A**) and anti-ghrelin antibody (FITC-Green; **Fig. 3B**) showed clear co-localization (Yellow; **Fig. 3C**) of nesfatin-1 and ghrelin immunoreactivity. Negative control is stained with only secondary antibodies alone **(** **Fig. 3D** **)**.

### NUCB2 protein expression in large intestine, small intestine and liver from male mice

**Figure 4:**
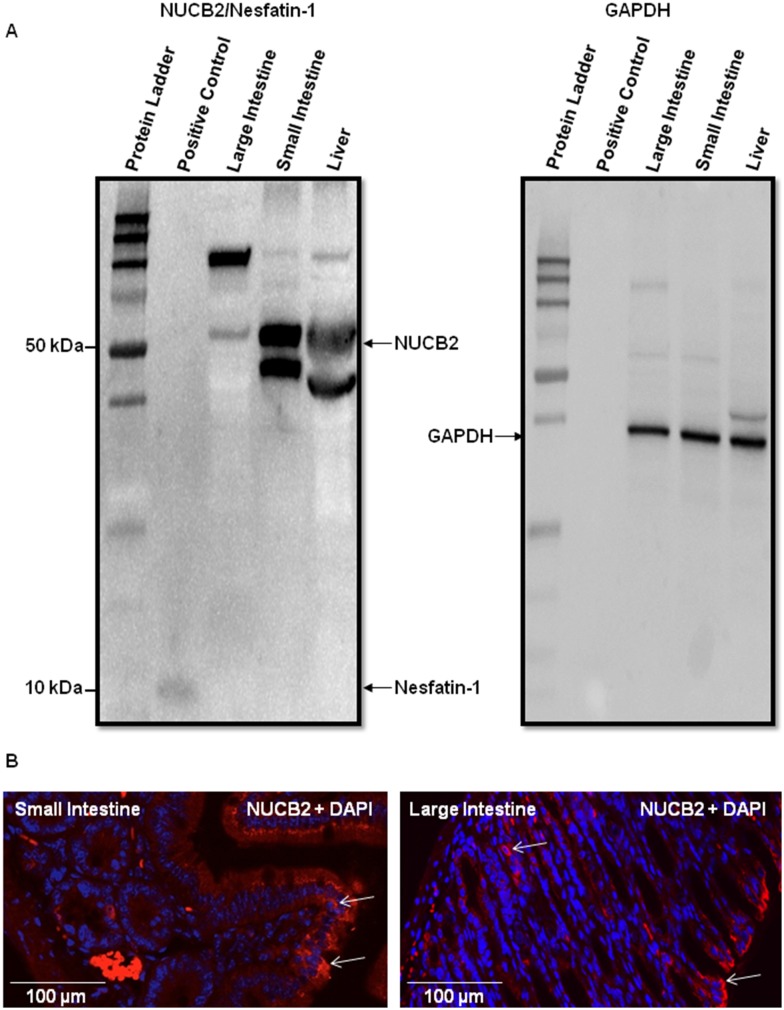
NUCB2 is expressed in the small intestine, large intestine and liver of male C57BL/6 mice. Tissues show distinct bands for NUCB2/nesfatin-1 corresponding to 50 kDa. Rat nesfatin-1 (Custom synthesized, ABGENT, 1 µg/µL) was used as a positive control which is shown as a distinct band corresponding to 10 kDa (A; Left Image). Mouse glyceraldehyde 3-phosphate dehydrogenase (GAPDH) is uniformly expressed in liver, small and large intestine. The blots that were loaded with 50 µg of total protein/well previously for NUCB2 protein detection were stripped and re-blocked with mouse GAPDH primary antibody. Representative blot (n = 4 independent experiments) showing distinct bands for GAPDH corresponding to 37 kDa (A; Right Image). Mucosal cells showing NUCB2/nesfatin-1 (Texas-Red) immunoreactivity, and DAPI are found in the small intestine (B; left image) and large intestine (B; right image) from male mice. Arrows point to cells that are immunopositive for NUCB2/nesfatin-1 and shows distinct DAPI-stained nuclei. Representative images were taken of 4 slides (8 sections/tissue) from three adult male mice. Scale bar = 100 µm.

NUCB2 protein is expressed in the large intestine, small intestine and liver from male mice showing distinct band for NUCB2 corresponding to approximately 50 KDa. Rat nesfatin-1 peptide used as a positive control is shown as a distinct band corresponding to approximately 10 kDa **(** **Fig. 4A** **; left image)**. However, no bands showing the fully processed nesfatin-1 were visible at 10 kDa in the tissue samples **(** **Fig. 4A** **; left image)**. We also found bands of approximately 47 kDa underneath the 50 kDa band in the small intestine and liver, but not in the large intestine **(** **Fig. 4A** **; left image)**. A distinct band for GAPDH used as the control house-keeping gene is observed at 37 kDa shown in all tissues **(** **Fig. 4A** **; right image)**. NUCB2/nesfatin-1 immunoreactivity is found in the mucosal cells of small intestine **(** **Fig. 4B** **; left image)** and large intestine **(** **Fig. 4B** **; right image)**.

### Effects of glucose and L-Tryptophan on NUCB2 mRNA expression in, and NUCB2/nesfatin-1 secretion from MGN3-1 cells

**Figure 5:**
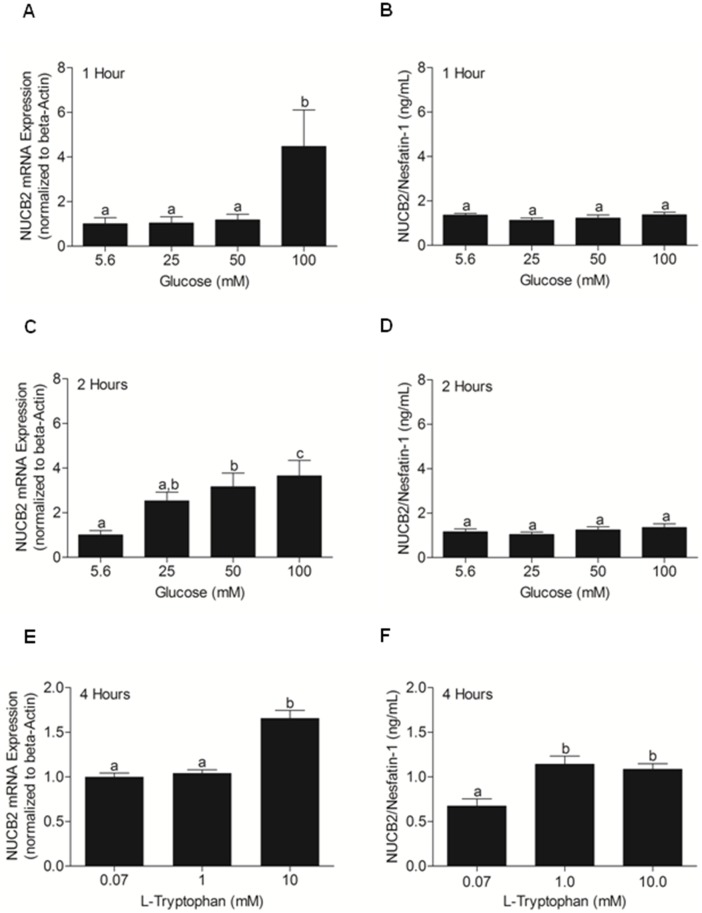
NUCB2 mRNA Expression (A, C, E) and NUCB2/nesfatin-1 Secretion (B, D, F) from MGN3-1 Cells Incubated with Different Concentrations of Glucose (5.6 mM, 25 mM, 50 mM and 100 mM) at Various Incubation Periods (1 Hour and 2 Hours), and with Different Concentrations of L-Tryptophan (0.07 mM, 1 mM, 10 mM; 4 Hours). MGN3-1 cells incubated at 100 mM glucose had a significant increase in NUCB2 mRNA expression at 1 hour post-incubation (A; p<0.05), but no significant differences was found in nesfatin-1 secreted into the media from the same cells (B). Similarly, glucose caused a dose dependent increase in NUCB2 mRNA expression at 2 hours post-incubation (C; p<0.5) without causing any changes in nesfatin-1 secretion (D). n = 9 wells/concentration pooled from 3 different studies. MGN3-1 cells incubated at 10 mM L-Tryptophan had a significant increase in NUCB2 mRNA expression than cells incubated at 0.07 mM and 1 mM L-Tryptophan (E; p<0.05). Nesfatin-1 secretion significantly increased from cells incubated at 1 mM and 10 mM L-Tryptophan than 0.07 mM L-Tryptophan (F; p<0.05). n = 12 wells/concentration pooled from 3 different studies. Letters b and c denote significant differences found between control (a) and various treatment groups, using One Way ANOVA followed by Tukey’s Multiple Comparison Test. There are no significant differences between groups marked by same letters.

Cells incubated at 100 mM glucose DMEM had a higher NUCB2 mRNA expression than cells incubated at 5.6, 25 and 50 mM DMEM glucose concentrations at 1 hour post-incubation **(** **Fig. 5A** **)**. At 2 hours post-incubation, cells incubated at 100 mM DMEM were significantly higher in NUCB2 mRNA expression than cells incubated at 5.6 and 50 mM glucose DMEM **(** **Fig. 5C** **)**. At 1 hour **(** **Fig. 5B** **)** and 2 hours **(** **Fig. 5D** **)**, there were no significant differences in NUCB2/nesfatin-1 secretion. NUCB2 mRNA expression was significantly higher in cells incubated at 10 mM L-Tryptophan **(** **Fig. 5E** **)** in comparison to cells incubated at 0.07 and 1.0 mM L-Tryptophan. NUCB2/nesfatin-1 secretion from cells incubated at 1.0 and 10.0 mM L-Tryptophan were significantly higher than cells incubated at 0.7 mM L-Tryptophan **(** **Fig. 5F** **)**.

### Effect of linolenic acid, octanoic acid and oleic acid on NUCB2 mRNA expression in, and NUCB2/nesfatin-1 secretion from MGN3-1 cells

**Figure 6:**
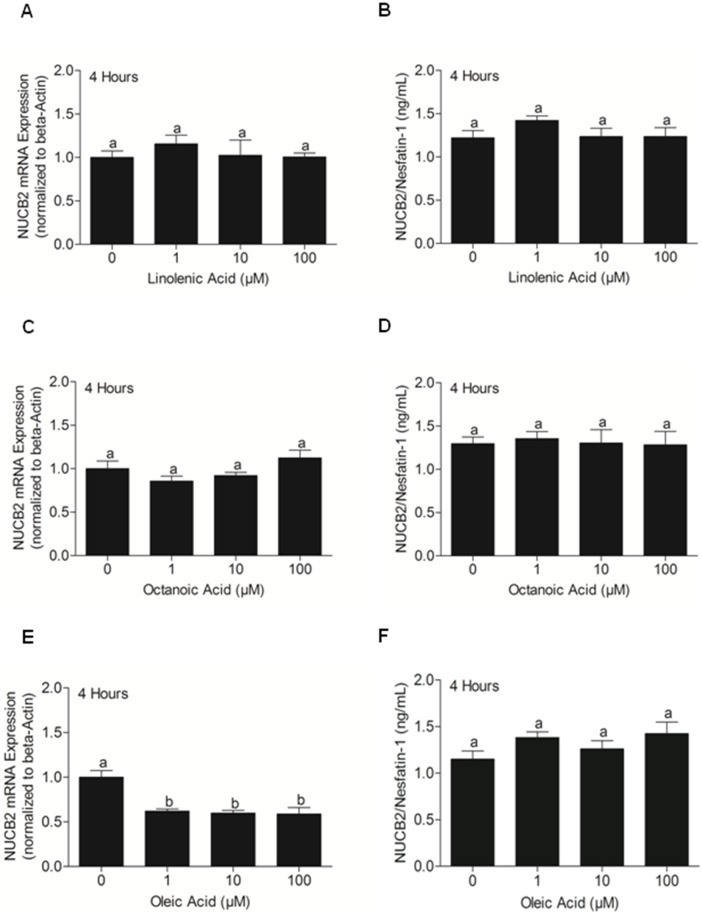
NUCB2 mRNA Expression (A, C, E) and NUCB2/nesfatin-1 Secretion (B, D, F) from MGN3-1 Cells Incubated for 4 Hours with Different Concentrations of Linolenic Acid, Octanoic Acid and Oleic Acid (0 µM, 1 µM, 10 µM, 100 µM). No change in NUCB2 mRNA expression (A, C) and nesfatin-1 secretion (B, D) when MGN3-1 cells were incubated with different concentrations of linolenic acid and octanoic acid. MGN3-1 cells incubated at 1, 10, 100 µM oleic acid had a significant decrease in NUCB2 mRNA expression (E; p<0.05), but no significant difference was found in nesfatin-1 secretion at 4 hours post-incubation (F; p<0.05). n = 9 wells/concentration pooled from 3 different studies. Different letters (a and b) shows significant differences found between control and various treatment groups, using One Way ANOVA followed by Tukey’s Multiple Comparison Test. There are no significant differences between groups marked by same letters.

We found NUCB2 mRNA expression significantly reduced in cells treated with 1, 10, and 100 µM oleic acid **(** **Fig. 6E** **)** in comparison to the control. No changes in NUCB2 mRNA were observed in cells treated with linolenic **(** **Fig. 6A** **)** and octanoic acid **(** **Fig. 6C** **)**. Further, NUCB2/nesfatin-1 secretion was unaltered in cells treated with different doses of linolenic acid **(** **Fig. 6B** **)**, octanoic acid **(** **Fig. 6D** **)**, and oleic acid **(** **Fig. 6F** **)**.

### Effect of L-tryptophan, linolenic acid, octanoic acid and oleic acid independently on ghrelin mRNA expression in, and total ghrelin secretion from MGN3-1 cells

**Figure 7:**
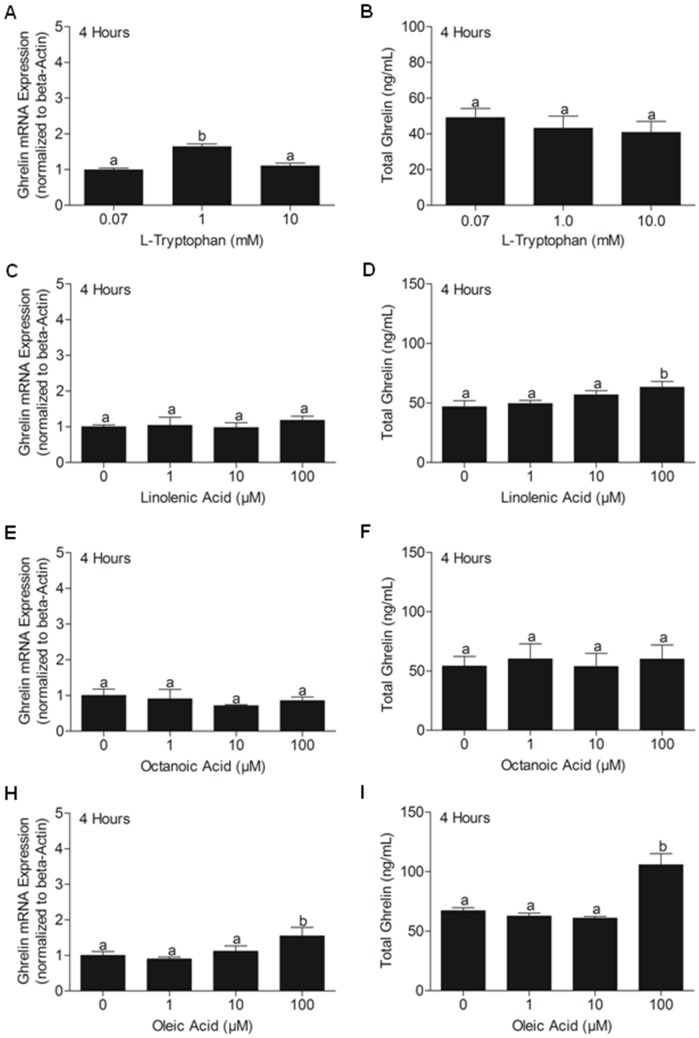
Ghrelin mRNA Expression (A, C, E, H) and Total Ghrelin Secretion (B, D, F, I) from MGN3-1 Cells Incubated for 4 Hours with Different Concentrations of L-Tryptophan (0.07 mM, 1 mM, 10 mM) and with Different Concentrations of Linolenic Acid, Octanoic Acid and Oleic Acid (0 µM, 1 µM, 10 µM, 100 µM). MGN3-1 cells incubated at 1 mM L-Tryptophan had a significant increase in Ghrelin mRNA expression than cells incubated at 0.07 mM and 10 mM L-Tryptophan (A; p<0.05). No change in total ghrelin secretion (B) when MGN3-1 cells were incubated with different concentrations of L-Tryptophan. n = 12 wells/concentration pooled from 3 different studies. No change in ghrelin mRNA expression (C), but a significant increase in total ghrelin secreted into the media from the same cells incubated at 100 µM linolenic acid (D; p<0.05). No change in ghrelin mRNA expression (E) and total ghrelin secretion (F) when MGN3-1 cells were incubated with different concentrations of octanoic acid. A significant increase in ghrelin mRNA expression (H; p<0.05) and total ghrelin secreted into the media from the same cells incubated at 100 µM oleic acid (I; p<0.05). n = 9 wells/concentration pooled from 3 different studies. Different letters shows significant differences found between control and various treatment groups, using One Way ANOVA followed by Tukey’s Multiple Comparison Test. There are no significant differences between groups marked by same letters.

No changes in ghrelin mRNA **(S1A Figure)** and total ghrelin secretion **(S1B Figure)** were found when cells were treated with different doses of glucose post 1 hour incubation. Ghrelin mRNA expression was significantly higher in cells incubated at 1 mM L-Tryptophan **(** **Fig. 7A** **)** in comparison to cells incubated at 0.07 and 10 mM L-Tryptophan. No significant difference in total ghrelin secretion from cells treated with different doses of L-Tryptophan **(** **Fig. 7B** **)**. We found no change in ghrelin mRNA expression **(** **Fig. 7C** **)**, but total ghrelin secretion was high from cells incubated at 100 µM linolenic acid **(** **Fig. 7D** **)** in comparison to control, 1 and 10 µM doses. No changes in ghrelin mRNA **(** **Fig. 7E** **)** and total ghrelin secretion **(** **Fig. 7F** **)** were found when cells were treated with different doses of octanoic acid. Meanwhile, ghrelin mRNA **(** **Fig. 7H** **)**, and total ghrelin secretion **(** **Fig. 7I** **)** were significantly higher in cells treated with 100 µM oleic acid, compared to the control, 1 and 10 µM oleic acid.

### Chronic effects of nutrients on NUCB2 mRNA expression and serum NUCB2/nesfatin-1 in mice

**Figure 8:**
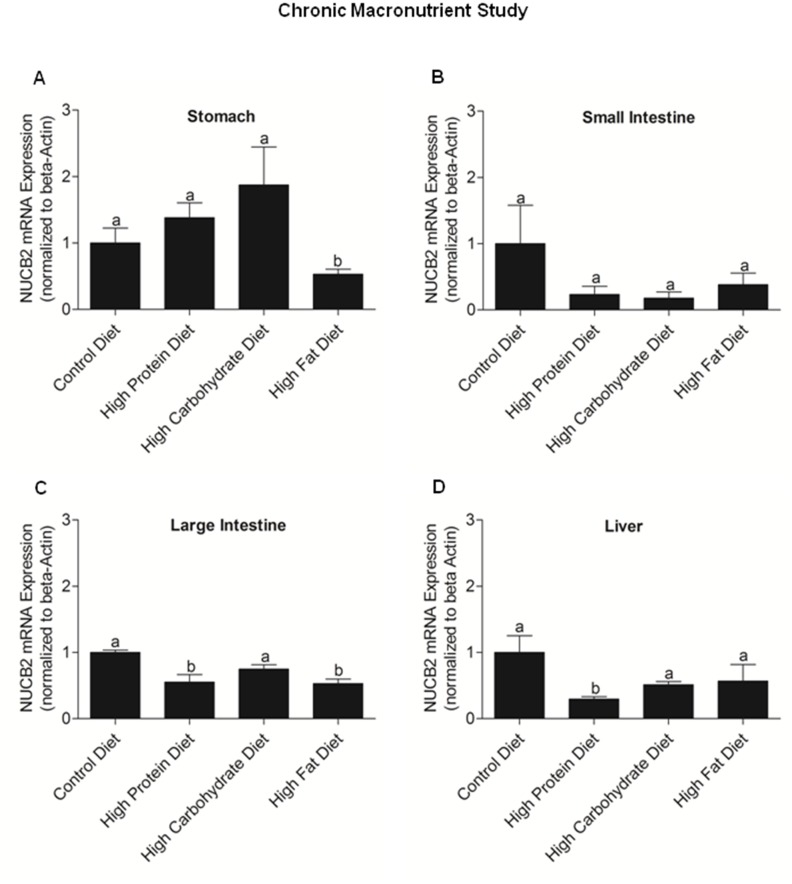
NUCB2 mRNA Expression in the Stomach (A), Small Intestine (Duodenum; B), Large Intestine (C) and Liver (D) of Mice Fed Various Diets. NUCB2 mRNA was significantly reduced in the stomach (A; p<0.05) from mice fed on a high fat diet than mice fed on the control, high protein and high carbohydrate diet (n = 5–6 mice/group). No significant difference were found in NUCB2 mRNA expression in the small intestine from mice fed on various diets [B; (CD; n = 5), (HP; n = 7), (HC; n = 7), (HF; n = 7)]. Mice fed on high protein and high fat diets had significantly low expression of NUCB2 mRNA in the large intestine (C; p<0.05) than mice fed on the control and high carbohydrate diet [(CD; n = 6), (HP; n = 7), (HC; n = 6), (HF; n = 7)]. Mice fed on a high protein diet had low expression of NUCB2 mRNA in the liver (D; p<0.05) than mice fed on other diets [(CD; n = 6), (HP; n = 7), (HC; n = 7), (HF; n = 7)]. Different letters (a and b) shows significant differences found between the various fed groups, using One Way ANOVA followed by Tukey’s Multiple Comparison Test. There are no significant differences between groups marked by same letters.

**Figure 9:**
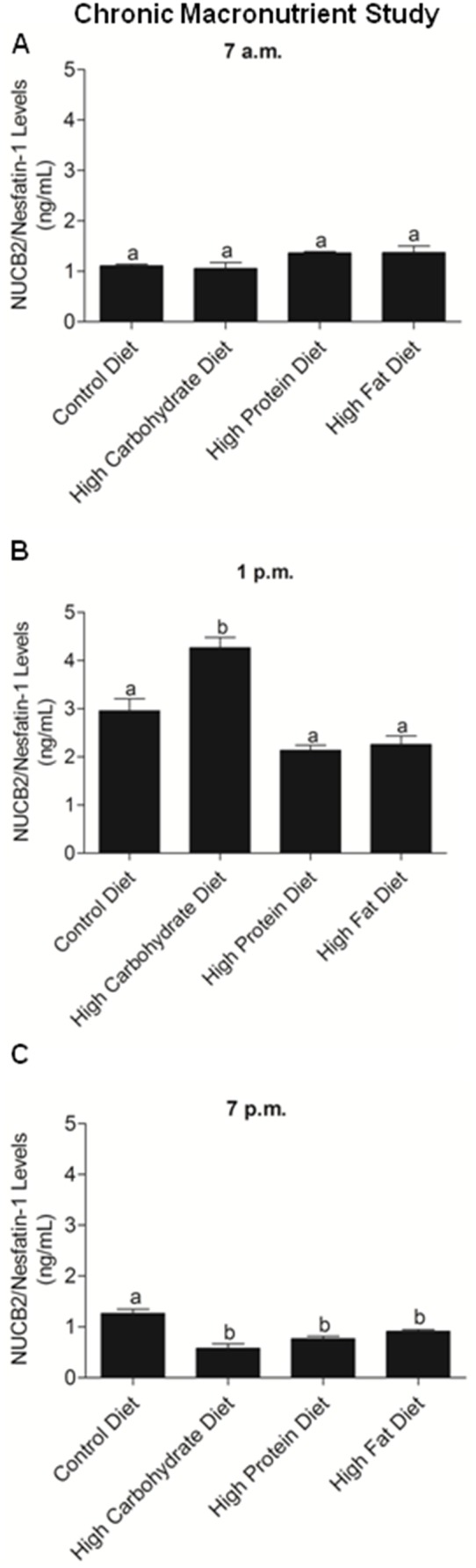
Serum NUCB2/Nesfatin-1 Levels at 7 a.m., 1 p.m., and 7 p.m. in Mice Fed Various Diets. Mice were chronically fed on a control diet (n = 6), high carbohydrate diet (n = 7), high protein diet (n = 7), and high fat diet (n = 7). No significant difference in serum NUCB2/nesatin-1 levels was found in mice fed on different diets at 7 a.m. (A). High carbohydrate diet fed mice had significantly higher serum NUCB2/nesatin-1 in circulation at 1 p.m. (B; p<0.05). Serum NUCB2/nesatin-1 was significantly lower in mice fed high carbohydrate, high protein or high fat, compared to control diet fed mice at 7 p.m. (C; p<0.05)**.** Different letters (a and b) shows significant differences found between the various fed groups, using One Way ANOVA followed by Tukey’s Multiple Comparison Test. There are no significant differences between groups marked by same letters.

The body weight, food intake and blood glucose profile of mice fed on various diets are shown in **S2 Figure**. Mice fed on a high fat diet had significantly low expression of NUCB2 mRNA in the stomach compared to those fed on a control, high protein or high carbohydrate diets **(** **Fig. 8A** **)**. There was no significant difference in the expression of NUCB2 mRNA in the small intestine between mice fed on a control, high protein, high carbohydrate or high fat diets **(** **Fig. 8B** **)**. Mice fed on the high protein and high fat diets have comparatively low expression of NUCB2 mRNA in the large intestine than mice fed on control or high carbohydrate diets **(** **Fig. 8C** **)**. Mice fed on a high protein diet had a significant low expression of NUCB2 mRNA in the liver than mice fed on the other diets **(** **Fig. 8D** **)**. At 7 a.m, there were no differences in serum nesfatin-1/NUCB2 levels in mice fed different diets **(** **Fig. 9A** **)**. At 1 p.m, the high carbohydrate diet fed mice had significantly higher serum nesfatin-1/NUCB2 in circulation **(** **Fig. 9B** **)**. Serum nesfatin-1/NUCB2 was significantly lower in mice fed high carbohydrate, high protein or high fat, compared to control diet fed mice at 7 p.m **(** **Fig. 9C** **)**.

### Acute effects of nutrients on NUCB2 mRNA expression, blood glucose and serum NUCB2/nesfatin-1 in mice

**Figure 10:**
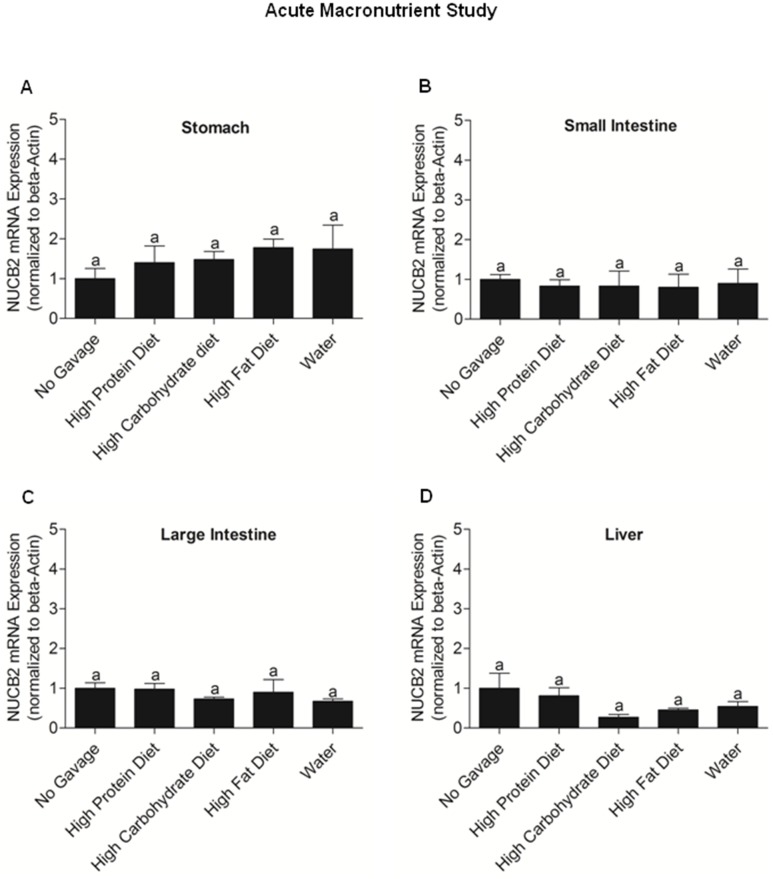
NUCB2 mRNA Expression in the Stomach (A), Small Intestine (Duodenum; B), Large Intestine (C) and Liver (D) of Mice gavaged with water, high protein, high carbohydrate, high fat and the No Gavage Group (n = 7 mice/group). No significant differences were found in NUCB2 mRNA in the stomach of mice gavaged with various liquid diets and no gavage group. Same letters (a) indicate no significant differences found between the various groups, using One Way ANOVA followed by Tukey’s Multiple Comparison Test.

**Figure 11:**
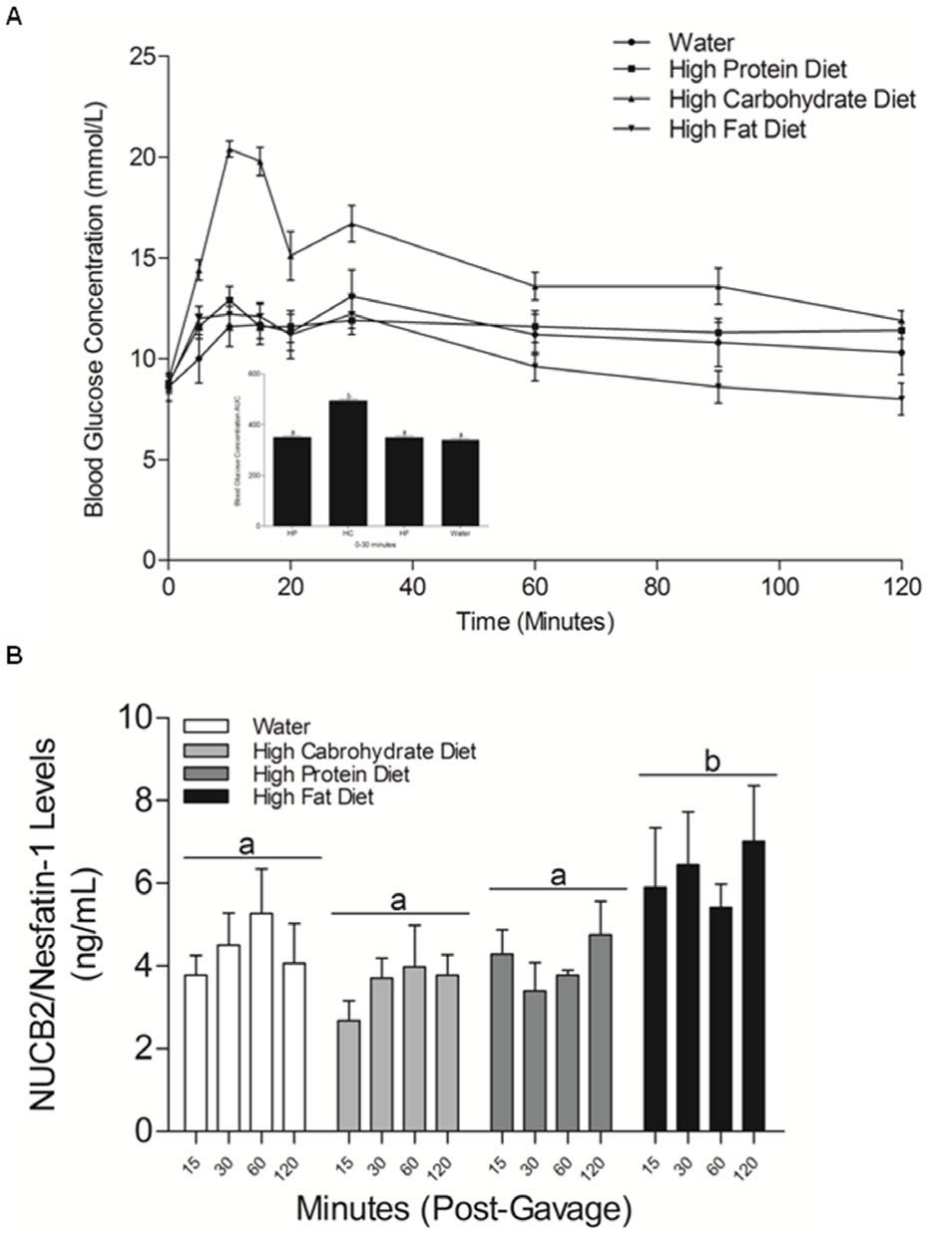
Blood Glucose and Serum NUCB2/Nesfatin-1 in mice during acute administration of various liquid diets and in no-gavage group. (**A**) Fold change in blood glucose measured during an oral gavage study in mice gavaged with a specific liquid diet, divided into 5 groups: high protein (n = 7), High fat (n = 7), High carbohydrate (n = 7), water (n = 7) and a no gavage group (n = 7). Mice gavaged with the high carbohydrate liquid diet had high concentrations of blood glucose compared to the no-gavage group and the groups gavaged with water, high protein and high fat liquid diets (p<0.05, ANOVA followed by Tukey’s Multiple Comparison Test). Blood glucose concentration - Area under the Curve from 0–30 measured during an oral gavage study in mice gavaged with a specific liquid diet, divided into 4 groups: high protein, high fat, high carbohydrate, and water (n = 7 mice/group). Letter *a* denotes no difference between the groups gavaged with water, high protein and high fat liquid diet. Letter b denotes that the high carbohydrate group had significantly elevated glucose levels compared to high protein, high carbohydrate, high fat and water gavaged mice during 0–30 minutes post-administration (p<0.05, One-Way ANOVA followed by Tukey’s Multiple Comparison Test). (**B**) Secretion profile representing circulating levels of NUCB2/Nesfatin-1 in mice gavaged with a specific liquid diet, divided into 4 groups: high protein, high fat, high carbohydrate, and water (n = 7 mice/group). Letter a denotes no difference in serum NUCB2/Nesfatin-1 levels between mice gavaged with water, high protein, high carbohydrate, high fat liquid diets and the no-gavage group within the four time points tested. Letter b denotes that the serum nesfatin-1/NUCB2 levels in the high fat diet fed group was significantly higher to the levels in the control mice or mice fed other diets at all time points (p<0.05, One-Way ANOVA followed by Tukey’s Multiple Comparison Test).

There were no significant changes in the expression of NUCB2 mRNA in the stomach **(** **Fig. 10A** **)**, small intestine **(** **Fig. 10B** **)**, large intestine **(** **Fig. 10C** **)** and liver **(** **Fig. 10D** **)** between mice fed on a high protein, high carbohydrate, high fat or water. We found that the group gavaged with the glucose diet had significant high blood glucose levels compared to the groups gavaged with water, high protein and high fat liquid diets **(** **Fig. 11A** **)**. In order to validate blood glucose tolerance in each group during the oral gavage study, we analyzed the area under the curve from 0–30 minutes. We found that the group gavaged with glucose had a significant increase in the concentration of blood glucose levels in the initial 30 minutes post-gavage. During the intra-peritoneal glucose tolerance test, NUCB2/nesfatin-1 levels in the high fat diet gavaged mice at 15, 30, 60, 120 minutes were significantly higher compared to mice fed with water, high protein and high carbohydrate diet, at all four time points tested **(** **Fig. 11B** **)**.

## Discussion

The first main contribution of this work is the characterization of MGN3-1 cells as a source of nesfatin-1 and a tool for studying the regulation of nesfatin-1 secretion. We found mRNAs encoding NUCB2, and its processing enzymes PC 1/3 and PC 2 in MGN3-1 cells. These findings are supported by NUCB2, PC 1/3 and PC 2 immunoreactivity detected in the same cells. Further, it was determined that MGN3-1 cells colocalize both nesfatin-1 and ghrelin, an observation that confirms previous findings of nesfatin-1 in the X/A ghrelin cells in the stomach (13). The cell line MGN3-1 used in this research is a pure population of ghrelinoma cells, and it has been characterized that these cells express prohormone convertases and ghrelin-O-acyl transferase [17], [18]. Our results, together with previous findings indicate that the enzyme machinery required to process NUCB2 to nesfatin-1 is present in MGN3-1 cells. We then utilized MGN3-1 cells to identify how nutrients modulate NUCB2 synthesis and secretion. Cells incubated for 2 hours at 100 mM glucose containing DMEM had significantly higher NUCB2 mRNA expression than cells incubated at lower glucose concentrations. However, we found that NUCB2/nesfatin-1 secretion was not influenced by changes in glucose concentrations introduced. Meanwhile, both NUCB2 mRNA expression and NUCB2/nesfatin-1 secretion were enhanced by L-Tryptophan. Since previous studies on nutrient regulation of hormones looked at the effects of fatty acids on protein secretion [19]–[22], we tried three fatty acids, oleic acid, linoleic acid and octanoic acid individually. Only oleic acid was found to inhibit NUCB2 mRNA expression, while the other two fatty acids were ineffective in modulating NUCB2 mRNA and NUCB2/nesfatin-1 secretion. Both in the glucose and oleic acid treatments, we found changes in NUCB2 mRNA expression, with no effects on nesfatin-1 secretion. It is possible that the NUCB2 mRNA expression and secretion in response to these nutrients are uncoupled in ghrelinoma cells. Further studies are required to elucidate the specific effectors in the transcriptional, translational and post-translational regulation of NUCB2/nesfatin-1 synthesis and secretion.

Glucose inhibits ghrelin secretion from stomach endocrine cells [36], [37]. In our studies using MGN3 cells, glucose was ineffective in causing any changes on ghrelin. Meanwhile, glucose elicited stimulatory effect on NUCB2 mRNA expression. The difference in our results is possibly due many difference in the experimental protocols, types of cells (primary cells versus cell lines) and doses used. Here, we also explored the effects of amino acids and fatty acids on ghrelin mRNA expression and secretion. As in the case of NUCB2 mRNA, the expression of ghrelin mRNA expression was also significantly higher in cells incubated with L-Tryptophan, but at a different dose (1 mM). Meanwhile, the effects of some fatty acids on ghrelin appear to be the opposite of what was seen on NUCB2 mRNA expression. Total ghrelin secretion from MGN3-1 cells incubated at 100 µM linolenic acid was significantly higher. In addition, ghrelin mRNA and total ghrelin secretion was significantly increased from cells incubated at 100 µM oleic acid. This data shows that fat stimulates ghrelin, while suppresses nesfatin-1. While nesfatin-1 is produced in stomach ghrelin cells, it has been shown that nesfatin-1 is present in distinct secretory vesicles [13]. Nutrient regulation of ghrelin secretion is also complex, with diverse effects in a tissues specific manner [33], [34] In general, proteins and lipids decreases ghrelin secretion [35]. Although the mechanisms by which nutrients are sensed by MGN3-1 cells are yet to be identified, at least in the case of fatty acids, that it may involve free fatty acid receptors. Other studies have identified that medium and long chain fatty acids bind to G-protein coupled receptors such as GPR40 and GPR120 [23], [24]. GPR40 is expressed in the brain and in the pancreatic β-cells and binds to free fatty acids stimulating the secretion of insulin [25]. Dietary fat has shown to stimulate cholecystokinin (CCK) by binding to GPR40 [26]. In contrast, GPR120 is expressed in the distal intestine, binding to long chain fatty acids such as α-linolenic acid [21]. Previous studies on the effects of free fatty acids in the enterendocrine cell line STC-1 have shown that fatty acids stimulate glucagon-like peptide-1 (GLP-1) and CCK [21], [27]. Further studies are required to unravel the mechanism of nutrient regulation of nesfatin-1 in gut cells.

A second major contribution of this work is that we determined NUCB2/nesfatin-1 mRNA, and protein (immunoreactivity) in the liver and small and large intestines of mice. The quantification of NUCB2 mRNA indicates that the relative abundance of NUCB2 is much lower in the intestine and liver, when compared to the stomach of mice. Approximately 50 kDa bands representing NUCB2 was present in total protein collected from the liver, and small and large intestines. However, no bands of the expected 10 kDa size representing the processed nesfatin-1 was detected in any of the tissues tested. This result concurs with previous findings of a full length NUCB2 protein in the liver, and small and large intestine of male rats and ICR mice [5]. Meanwhile, Stengel and colleagues [13], were unable to find NUCB2/nesfatin-1 in the liver and intestinal samples collected from male Sprague Dawley rats. This inability to detect NUCB2 in Sprague Dawley rats is unclear. Possible reasons include species differences in NUCB2 expression and/or differences in the physiological status of the rats used in that study. Similar to both [5], [13] studies discussed above, we were unable to find the processed nesfatin-1 in any of the tissues tested. Stengel and colleagues [13] also found a processed band of approximately 47 kDa in their Western blot. Similar to this, we also detected second band 3 kDa less in molecular weight (approximately 47 kDa) in the small intestine and liver, but not in the large intestine. As concluded by Stengel et al., [13], the 47 kDa band suggests that the signal peptide in the NUCB2 from liver and small intestine could be cleaved, and the precursor released into circulation. The mucosal cells of both small and large intestine showed sparsely distributed cells that are immunopositive for NUCB2/nesfatin-1. Whether the nesfatin-1 positive cells are indeed intestinal enteroendocrine cells, and nesfatin-1 actions on intestinal physiology require further assessment.

Mice fed on a high fat diet had significantly lower expression of NUCB2 mRNA in the stomach compared to mice fed on the other diets. This was also evident in our *in vitro* studies where MGN3-1 gastric cells had low NUCB2 mRNA expression levels when treated with oleic acid. The difference in the levels of NUCB2 mRNA expression could be a result of how various gastric cells absorb nutrients in response to chronic intake of carbohydrate and fats. The process by which how fats and carbohydrates are processed by gastric mucosal cells likely also affects endogenous nesfatin-1. Central administration of nesfatin-1 inhibits gastric acid secretion [32]. It would be interesting to investigate enzymes that are involved in the digestion of carbohydrates and fat including pancreatic amylase and lipase to determine whether they are modulated simultaneously with NUCB2 mRNA expression in the stomach. We found no significant difference in NUCB2 mRNA expression in the small intestine in all the four diet fed groups. But in the large intestine, we found that mice fed on the high protein and high fat diet had comparatively low expression of NUCB2 mRNA than mice fed on the control and high carbohydrate diet. NUCB2/nesfatin-1 may be involved in the absorption of nutrients as well as controlling the secretion of intestinal hormones [5]. Other studies report NUCB2/nesfatin-1 immunoreactivity is also observed in the Brunner’s glands of SD rats and ICR mice [5]. These glands are involved in secreting alkaline products such as bicarbonate, mucus containing intestinal lipase, peptidase that could be influenced by nutrient absorption [28] affecting NUCB2 gene expression.

We found that high protein fed mice had significantly lower NUCB2 mRNA expression in the liver, compared to mice fed other diets. Previous studies have shown that nesfatin-1 is a glucose dependent insulinotropic peptide [4]. Liver is a major insulin responsive tissue involved in glucose homeostasis. It has been shown that peripheral infusion of nesfatin-1 upregulates mRNAs encoding phosphoenolpyruvate carboxykinase 1 and glucose-6-phosphatase, two enzymes that are critical in hepatic gluconeogenesis in the liver [30]. The endogenous changes in nesfatin-1 due to nutrient abundance might result in local changes in other liver proteins involved implicated in glucose metabolism. We found no changes in NUCB2/nesfatin-1 levels in mice, when sampled at 7 a.m, the onset of light phase. However, at 1 p.m, the mice fed high carbohydrate have the highest levels of nesfatin-1. At 7 p.m, all mice, expect the ones in the control group had significantly lower nesfatin-1 levels. Such suppression of an anorexigen prior to the onset of feeding time is also supportive of the satiety role of endogenous nesfatin-1. This difference in circulating NUCB2/nesfatin-1 levels could be attributed to a possible circadian pattern of NUCB2/nesfatin-1 release. In the acute diet study, nutrients had no significant differential effects in the expression of NUCB2 mRNA in the stomach, small intestine, large intestine and liver of mice. As expected, the acute administration of a high carbohydrate diet increased blood glucose levels within the initial 30 minutes. However, no significant increase in NUCB2/nesfatin-1 levels in the glucose gavaged group was found. Meanwhile, NUCB2/nesfatin-1 levels at 15, 30, 60, and 120 minutes post-gavage were significantly increased in mice fed on a high fat liquid diet. These results indicate that the nutrient elicited changes in NUCB2 expression varies depending on the mode of treatment and the duration of experiment.

## Conclusions

We provide the first set of information on nutrient regulation of nesfatin-1. Our findings suggest that the effects of diets on the expression of endogenous NUCB2/nesfatin-1 are myriad, with specific effects on mRNA expression versus secretion, in a dose and time dependent manner. We characterised MGN3-1 cells as nesfatin-1 secreting cells with the NUCB2 processing machinery, suggesting that this cell line is useful for studying nesfatin-1 biology. Glucose simulates NUCB2 mRNA expression in a dose and time–dependent manner in MGN3-1 cells. We also found that L-Tryptophan stimulates NUCB2 mRNA expression and nesfatin-1 secretion. From our *in vivo* studies, NUCB2 mRNA expression was significantly lower in the liver of mice fed on a high protein diet compared to mice fed other diets. High fat fed mice had a significant reduction in NUCB2 mRNA expression in the stomach, while high protein and high fat diet resulted in the attenuation of NUCB2 mRNA in the large intestine. Mice fed on the high protein, high carbohydrate and high fat demonstrated a post-prandial increase in NUCB2/nesfatin-1 secretion. Our results indicate that the synthesis and secretion of nesfatin-1 are altered by relative amount of nutrients and such effects are dependent on the amount of nutrients, tissues and time of the day or duration of treatment. While such variations exist, our data in general support that fat is inhibitory, while carbohydrate and protein are nesfatin-1 stimulatory. Nesfatin-1 is explored as an anti-obesity compound. Nutrient dependent changes in endogenous NUCB2/nesfatin-1 should also be considered, especially when developing diet or exogenous nesfatin-1 based potential therapies for obesity and related metabolic diseases.

## Supporting Information

S1 Figure **Ghrelin mRNA Expression (A) and Total Ghrelin Secretion (B) from MGN3-1 Cells Incubated for 1 Hour with Different Concentrations of Glucose (5.6 mM, 25 mM, 50 mM and 100 mM).** No changes in ghrelin mRNA and total ghrelin secretion were found when cells were treated with different doses of glucose post 1 hour incubation.
(TIF)

S2 Figure **Weekly Body Weight (A), Blood Glucose (B) and Food Intake to Body Weight Ratio (C) on Mice Fed Chronically on Various Nutrient Diets for 17 Weeks.** Mice fed on a control diet and a high fat diet was had an increase in the body weight than mice fed with a high protein and a high carbohydrate diet (A; p<0.05). Mice fed on a high protein diet and high carbohydrate diet had lower weekly blood levels than mice fed a control and high fat diet (B; p<0.05). Mice fed on a control diet and a high fat diet was had an increase in the ratio of food intake to body weight than mice fed with a high protein and a high carbohydrate diet (C; p<0.05). Mice (n = 6–7 mice/group) had *ad libitum* access to water and their specific diet, control diet, high carbohydrate diet, high protein diet, and high fat diet. Significant difference was found between the various fed groups, using One Way ANOVA followed by Tukey’s Multiple Comparison Test.
(TIF)
