# Supplementary figures and images for: Nutrients Differentially Regulate Nucleobindin-2/Nesfatin-1 In Vitro in Cultured Stomach Ghrelinoma (MGN3-1) Cells and In Vivo in Male Mice
Source: PLoS One. 2014 Dec 15;9(12):e115102. doi: 10.1371/journal.pone.0115102 (PMC4266631; doi:10.1371/journal.pone.0115102)

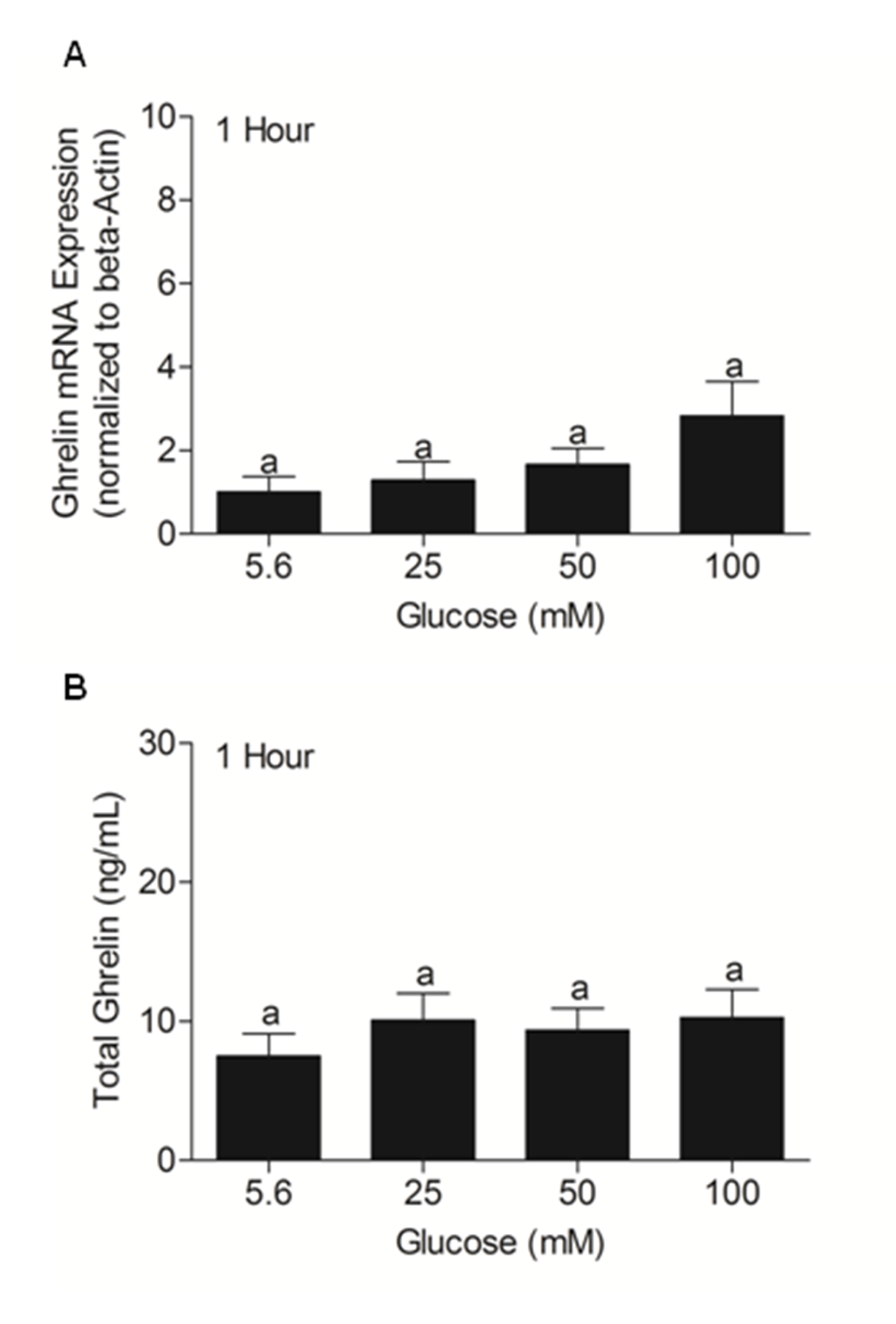

Supplement: S1 Figure — Ghrelin mRNA Expression (A) and Total Ghrelin Secretion (B) from MGN3-1 Cells Incubated for 1 Hour with Different Concentrations of Glucose (5.6 mM, 25 mM, 50 mM and 100 mM). No changes in ghrelin mRNA and total ghrelin secretion were found when cells were treated with different doses of glucose post 1 hour incubation. (TIF) [file pone.0115102.s001.tif]

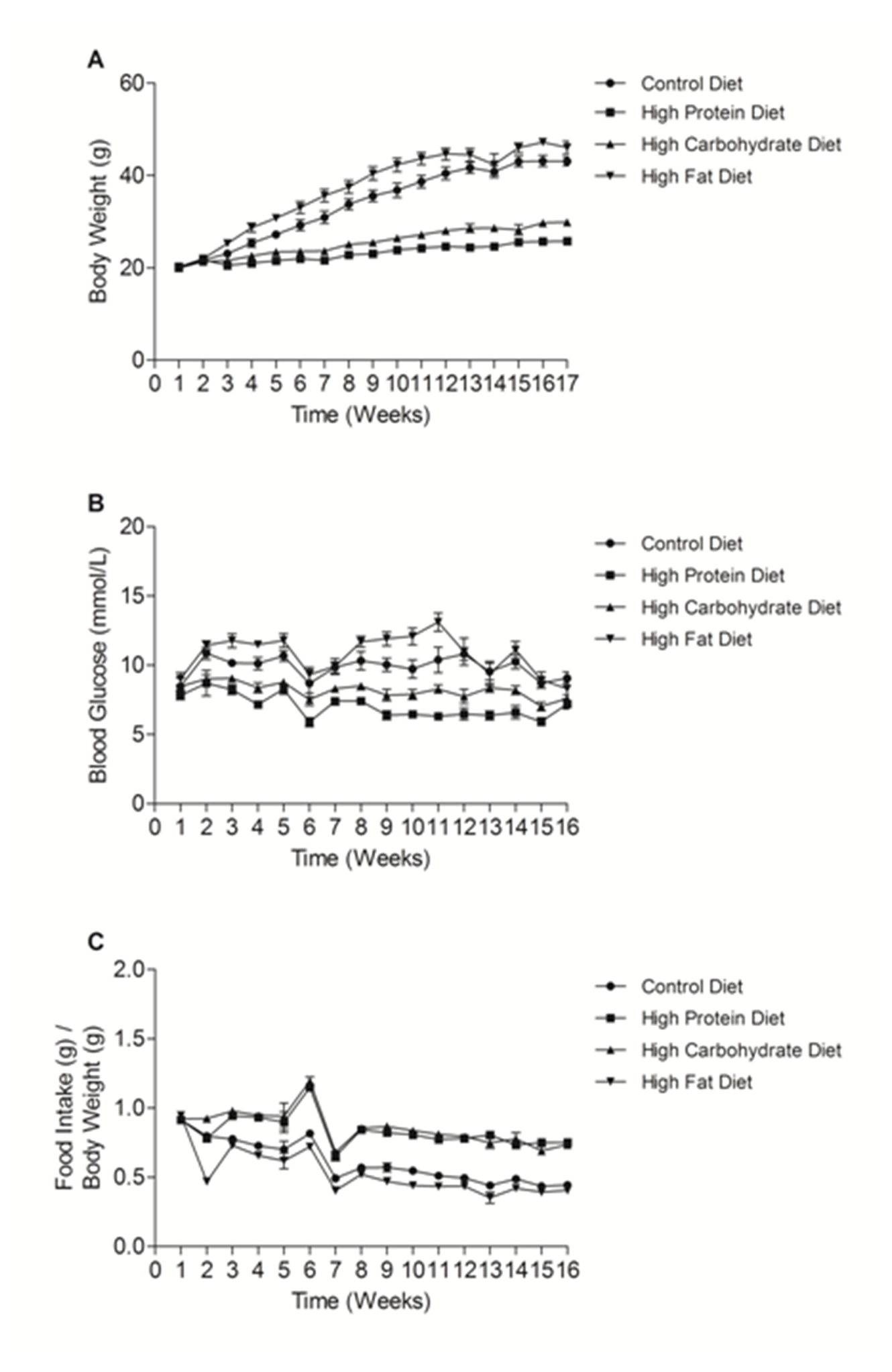

Supplement: S2 Figure — Weekly Body Weight (A), Blood Glucose (B) and Food Intake to Body Weight Ratio (C) on Mice Fed Chronically on Various Nutrient Diets for 17 Weeks. Mice fed on a control diet and a high fat diet was had an increase in the body weight than mice fed with a high protein and a high carbohydrate diet (A; p<0.05). Mice fed on a high protein diet and high carbohydrate diet had lower weekly blood levels than mice fed a control and high fat diet (B; p<0.05). Mice fed on a control diet and a high fat diet was had an increase in the ratio of food intake to body weight than mice fed with a high protein and a high carbohydrate diet (C; p<0.05). Mice (n = 6–7 mice/group) had ad libitum access to water and their specific diet, control diet, high carbohydrate diet, high protein diet, and high fat diet. Significant difference was found between the various fed groups, using One Way ANOVA followed by Tukey’s Multiple Comparison Test. (TIF) [file pone.0115102.s002.tif]
